# Integrated analysis of 1804 samples of six centers to construct and validate a robust immune-related prognostic signature associated with stromal cell abundance in tumor microenvironment for gastric cancer

**DOI:** 10.1186/s12957-021-02485-y

**Published:** 2022-01-05

**Authors:** Junyu Huo, Ge Guan, Jinzhen Cai, Liqun Wu

**Affiliations:** Liver Disease Center, The Affiliated Hospital of Qingdao University, No. 59 Haier Road, Qingdao, 266003 China

**Keywords:** Gastric cancer, Immune, Stromal cells, Prognostic, Signature, Tumor microenvironment

## Abstract

**Background:**

Stromal cells in tumor microenvironment could promote immune escape through a variety of mechanisms, but there are lacking research in the field of gastric cancer (GC).

**Methods:**

We identified differential expressed immune-related genes (DEIRGs) between the high- and low-stromal cell abundance GC samples in The Cancer Genome Atlas and GSE84437 datasets. A risk score was constructed basing on univariate cox regression analysis, LASSO regression analysis, and multivariate cox regression analysis in the training cohort (*n*=772). The median value of the risk score was used to classify patients into groups with high and low risk. We conducted external validation of the prognostic signature in four independent cohorts (GSE26253, *n*=432; GSE62254, *n*=300; GSE15459, *n*=191; GSE26901, *n*=109) from the Gene Expression Omnibus (GEO) database. The immune cell infiltration was quantified by the CIBERSORT method.

**Results:**

The risk score contained 6 genes (AKT3, APOD, FAM19A5, LTBP3, NOV, and NOX4) showed good performance in predicting 5-year overall survival (OS) rate and 5-year recurrence-free survival (RFS) rate of GC patients. The risk death and recurrence of GC patients growing with the increasing risk score. The patients were clustered into three subtypes according to the infiltration of 22 kinds of immune cells quantified by the CIBERSORT method. The proportion of cluster A with the worst prognosis in the high-risk group was significantly higher than that in the low-risk group; the risk score of cluster C subtype with the best prognosis was significantly lower than that of the other two subtypes.

**Conclusion:**

This study established and validated a robust prognostic model for gastric cancer by integrated analysis 1804 samples of six centers, and its mechanism was explored in combination with immune cell infiltration characterization.

## Background

Tumor microenvironment (TME) is a mixture of fluid, immune cells, stromal cells, and blood vessels that wrap the tumor [1]. Stromal cells are the main component of TME, including angiogenic vascular cell (AVC), cancer-associated fibroblast (CAF), and cancer-associated adipocyte cell (CAA) [2], which is closely related to the occurrence, development, invasion, and metastasis of the tumor [3, 4]. Therefore, stromal cells have been paid growing attention as a potential therapeutic target to effectively inhibit the progression of cancer [5–7].

The morbidity and mortality of gastric cancer (GC) have always been in the forefront of malignant tumors [8, 9]. Most patients with GC are in advanced stage when they are diagnosed, which leads to poor prognosis of GC after surgery [10]. In recent years, with the emergence of new treatments such as targeted therapy and immunotherapy [11, 12], the overall prognosis of GC has been greatly improved, but it is still unsatisfactory, and the conventional TNM staging is difficult to accurately evaluate the prognosis of GC after surgery, so developing an effective prognostic evaluation scheme has always been a research hotspot in the field of GC.

The immune response of tumor is an important process in the occurrence and development of tumor [13]. Without immune monitoring, the malignant biological behavior of tumor will be further accelerated, thus promoting the proliferation, invasion, and metastasis of tumor [14]. The latest study found that the synergistic action of stromal cells and immune cells makes TME appear as an immunosuppressive microenvironment, which provides favorable conditions for the growth and immune escape of tumor cells [15–17]. Therefore, understanding the interaction between stromal cells and immunophenotype plays is expected to find new targets to curb tumor progression.

This study explored the relationship between stromal cells and immune-related genes, established and validated a robust prognostic signature for GC by intergrated analysis of 1804 samples of six centers.

## Materials and methods

### Data acquisition

**Fig. 1:**
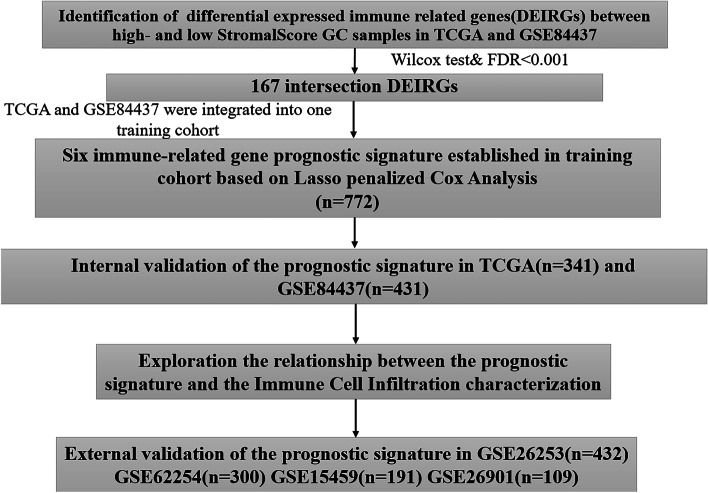
The workflow chart of this study

We obtained the gene expression profiles and corresponding clinical data from The Cancer Genome Atlas (TCGA, https://portal.gdc.cancer.gov/) and the Gene Expression Omnibus (GEO) database (GSE84437, https:// www.ncbi.nlm.nih.gov/geo/). There were 341 and 431 GC patients with completed prognostic information in the TCGA and the GSE84437 dataset respectively. The gene expression profiles and corresponding clinical data of the four independent cohorts (GSE26253, *n*=432; GSE62254, *n*=300; GSE15459, *n*=191; GSE26901, *n*=109) were downloaded from the GEO database. During the process of data acquisition, we complied with the access principles of TCGA and GEO database. The data analyzed in our work were acquired from public databases; the approval from the local ethics committee was not needed. The workflow of this study is shown in Fig. 1.

### Estimation stromal cells abundance in tumor microenvironment of TCGA and GSE84437 dataset

ESTIMATE (Estimation of STromal and Immune cells in MAlignant Tumor tissues using Expression data) is a tool for predicting tumor purity based on single sample Gene Set Enrichment Analysis, and the presence of infiltrating stromal/immune cells in tumor tissues using gene expression data [18]. Stromal score generated from ESTIMATE algorithm captures the presence stroma in tumor tissue; generally, the higher the stromal score, the higher the stromal cell abundance in tumor tissue. We calculated the stromal score of the TCGA and GSE84437 dataset using the R package “estimate”. Patients in the two independent cohorts were divided into high- and low-stromal score groups according to the median value of stromal score respectively, the Kaplan-Meier survival analysis was used to compare the overall survival between the two groups, and the *p* value of log-rank test <0.05 was suggested to be statistical significance.

### Identification of StromalScore Associated Immune-Related Genes (SAIRGs)

The immune-related gene list was obtained from the ImmPort database (https://immport.niaid.nih.gov). We extracted the immune-related genes from the TCGA and GSE84437 dataset, respectively, and identified the differentially expressed immune-related genes (DEIRGs) between the high- and low-stromal score group with Wilcoxon rank-sum test in “limma” R package in the TCGA and GSE84437 dataset. A false discovery rate (FDR) of < 0.001 was considered to be significant. The intersection DEIRGs of the two datasets were regarded as SAIRGs.

### Development and validation of an immune-related gene prognostic signature

We used combat in R package “sva” to remove the batch effects between the TCGA and GSE84437 datasets and merged them into a training cohort contained 772 patients. The univariate Cox regression analysis was used for screening the prognostic related SAIRGs with *p*<0.05. The least absolute shrinkage and selection operator (LASSO) algorithm was applied to remove the overfitting between prognostic related genes with penalty parameter tuning performed via 10-fold cross-validation. We subsampled the dataset 1000 times and selected the genes over 900 repeated times while the LASSO penalized Cox analysis was implemented [19]. A subselection of genes was detected as a result of a penalty proportional to their size to shrink the regression coefficient [19]. Only genes with nonzero regression coefficients were retained for subsequent multivariate Cox regression analyses. A risk score was constructed using regression coefficients derived from multivariate Cox regression analysis of each gene multiple the expression level of each gene [20, 21]. The GC patients were classified into low-risk and high-risk groups considering the median risk score. The LASSO regression analysis was performed with “glmnet” R package. The Kaplan–Meier survival curve and the time depend ROC curve were generated with the R package “survminer” and “survivalROC” respectively to assess the performance of the risk score. The risk score’s performance was conducted external validation in four independent cohorts (GSE26253, *n*=432; GSE62254, *n*=300; GSE15459, *n*=191; GSE26901, *n*=109).

### Immune cell infiltration (ICI) characterization

CIBERSORT is an analytical tool from the Alizadeh Lab developed by Newman et al. to provide an estimation of the abundances of member cell types in a mixed cell population, using gene expression data [22]. The 22 kinds of immune cell infiltration (ICI) proportions were quantified by the CIBERSORT method in this research; the samples with high prediction accuracy (*p* < 0.05) of prediction were considered for subsequent analysis [23].We conducted cluster analysis based on the R-package “consensusclusterplus” according to the results of ICI.

## Results

### The higher stromal score associated with poor prognosis of GC

**Fig. 2:**
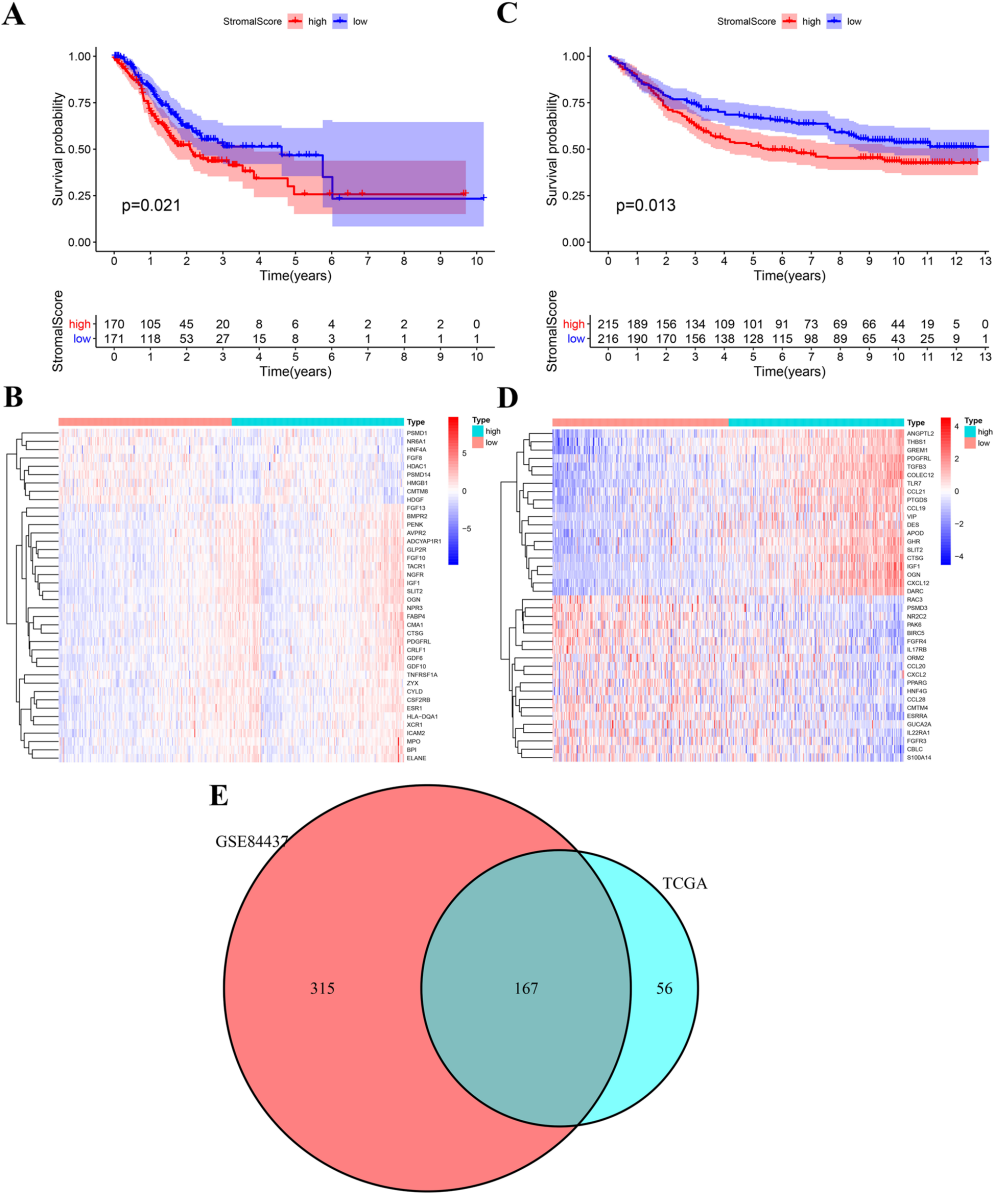
Identification of StromalScore Associated Immune-Related Genes (SAIRGs). **A** The Kaplan–Meier survival analysis regarding stromal score and OS in the TCGA cohort. **B** The heatmap of differential expressed immune-related genes (DEIRGs) between the high- and low-stromal score in the TCGA cohort. **C** The Kaplan–Meier survival analysis regarding stromal score and OS in the GSE84437 cohort. **D** The heatmap of differentially expressed immune-related genes (DEIRGs) between the high- and low-stromal score in the GSE84437 cohort. **E** The Venn plot of intersection genes

The GC patients with higher stromal score who had unfavorable clinical outcome were observed in the two large sample and independent cohorts (Fig. 2A, C). Next, we identified the DEIRGs between the high- and low-stromal score group in the TCGA and GSE84437 respectively (Fig. 2B, D). A total of 167 intersection DEIRGs were retained the subsequent analysis (Fig. 2E).

### Identification of prognostic related DEIRGs

**Fig. 3:**
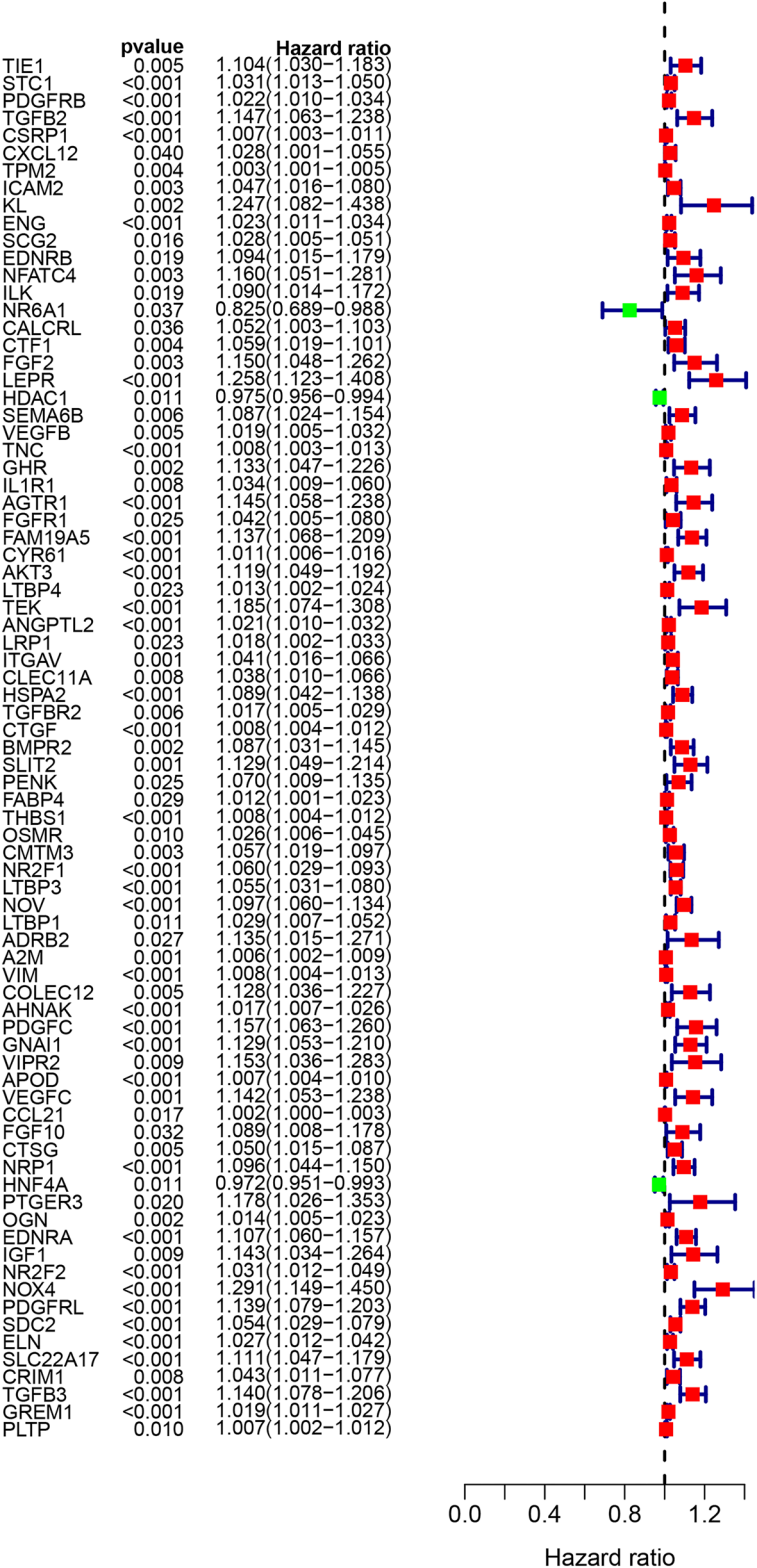
The forest plot of prognostic related genes identified by the univariate Cox analysis

The TCGA and GSE84437 datasets were intergrated into one large sample training cohort (*n*=772) for the establishment of the prognostic model. By univariate Cox regression analysis, we screened 79 prognostic related genes from the 167 intersection DEIRGs (Fig. 3).

### Construction of a six-immune gene prognostic signature in the training cohort

**Fig. 4:**
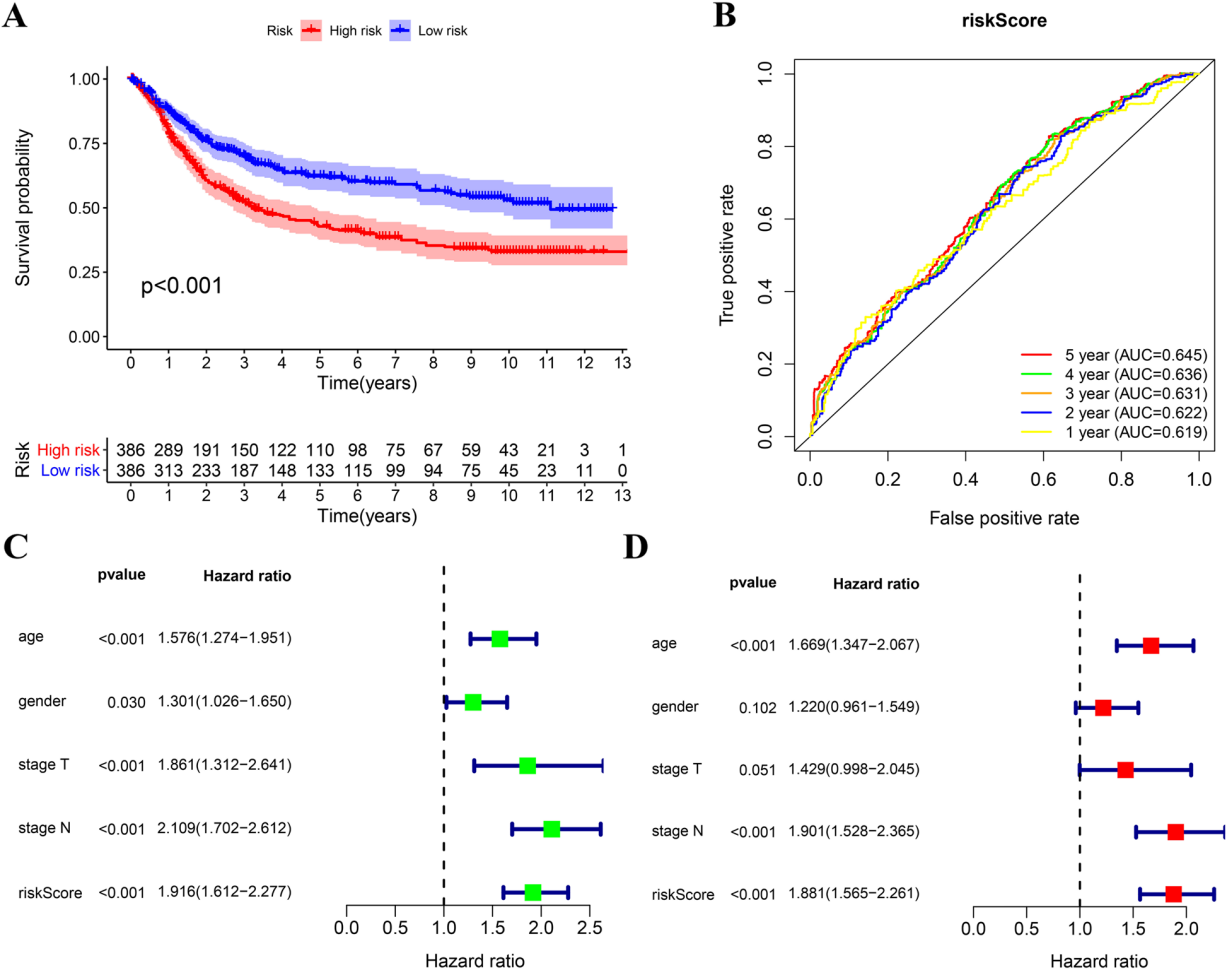
Prognostic assessment of the risk score in the training cohort. **A** The Kaplan–Meier survival analysis regarding risk score and OS in the training cohort. **B** The time-dependent ROC analysis of the risk score for predicting the OS of patients in the training cohort. **C** The forest plot of the univariate Cox analysis. **D** The forest plot of the multivariate Cox analysis

A total of six genes were retained to construct the risk score by LASSO regression analysis and multivariate Cox regression analysis. The risk score = AKT3* 0.05467+ APOD* 0.002885+ FAM19A5* 0.05842+ LTBP3* 0.02132+ NOV* 0.0529+ NOX4* 0.1367. According to the median value, 386 patients with risk score greater than 0.9438 were assigned into the high risk group, and the remaining 386 patients with risk score less than 0.9438 were assigned into the low risk group. The patients in the high risk group showed significantly reduced overall survival (OS) compared to the low risk group (Fig. 4A). The area under the curve (AUC) values for the risk score predicting OS at 1, 3, and 5 years were 0.619, 0.631, and 0.645 respectively (Fig. 4B). As suggested by the univariate and multivariate Cox regression analysis, the risk score could be served as an independent prognostic risk factor (Fig. 4C, D).

### The prognostic signature was applicable for patients with different clinical features

**Fig. 5:**
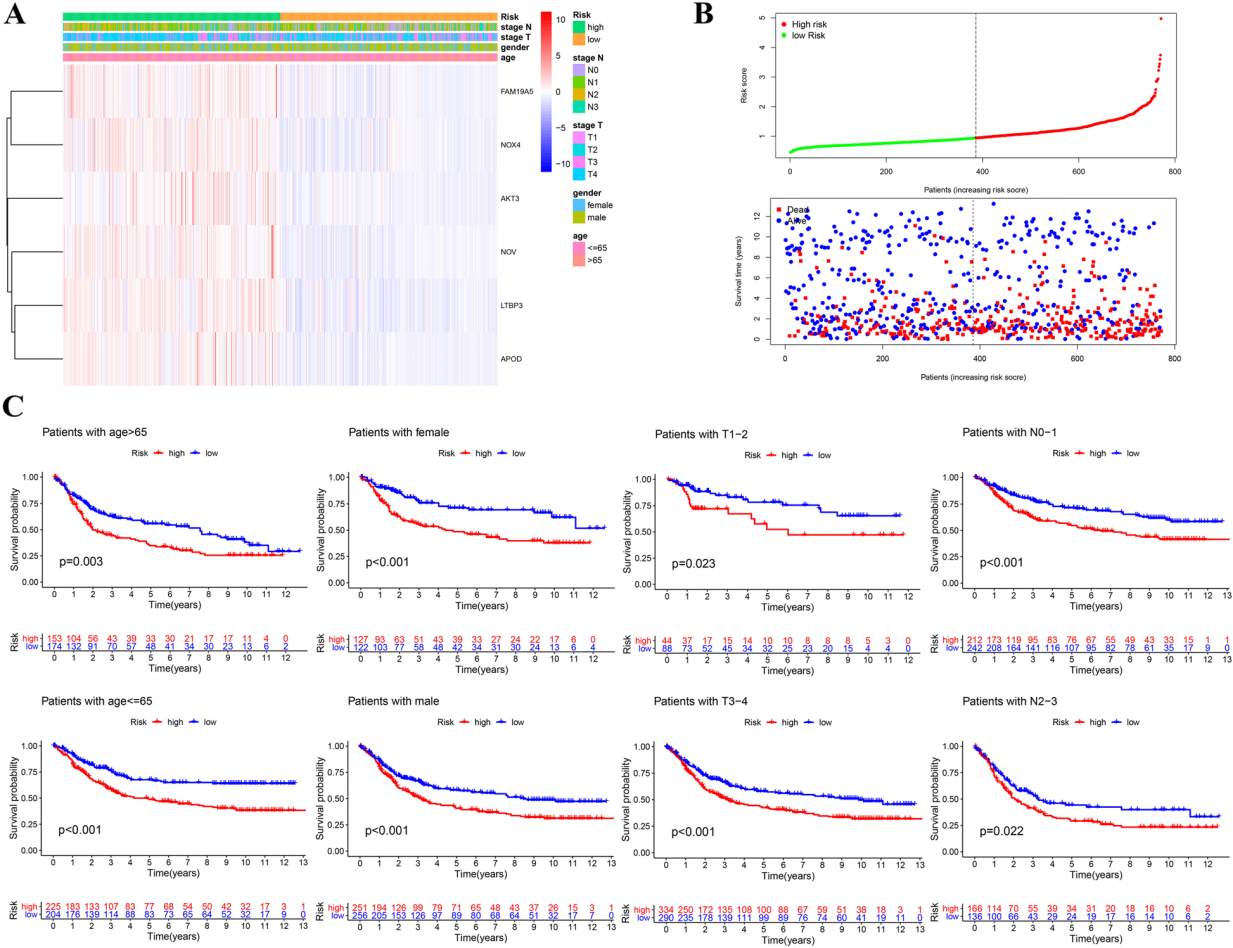
Clinical subgroup validation of the prognostic risk score. **A** The heatmap of six genes. **B** The risk score distribution and the survival status of patients in the training cohort. **C** Clinical subgroup survival analysis

The six genes of the signature were all upregulated in the high risk group (Fig. 5A). The risk death of GC patients growing with the increasing risk score (Fig. 5B). The poor prognosis of high risk group in each clinical subgroup demonstrated the prognostic signature was suitable for patients with different clinical characteristics (Fig. 5C).

### Internal validation of the prognostic signature in the TCGA and GSE84437 cohort

**Fig. 6:**
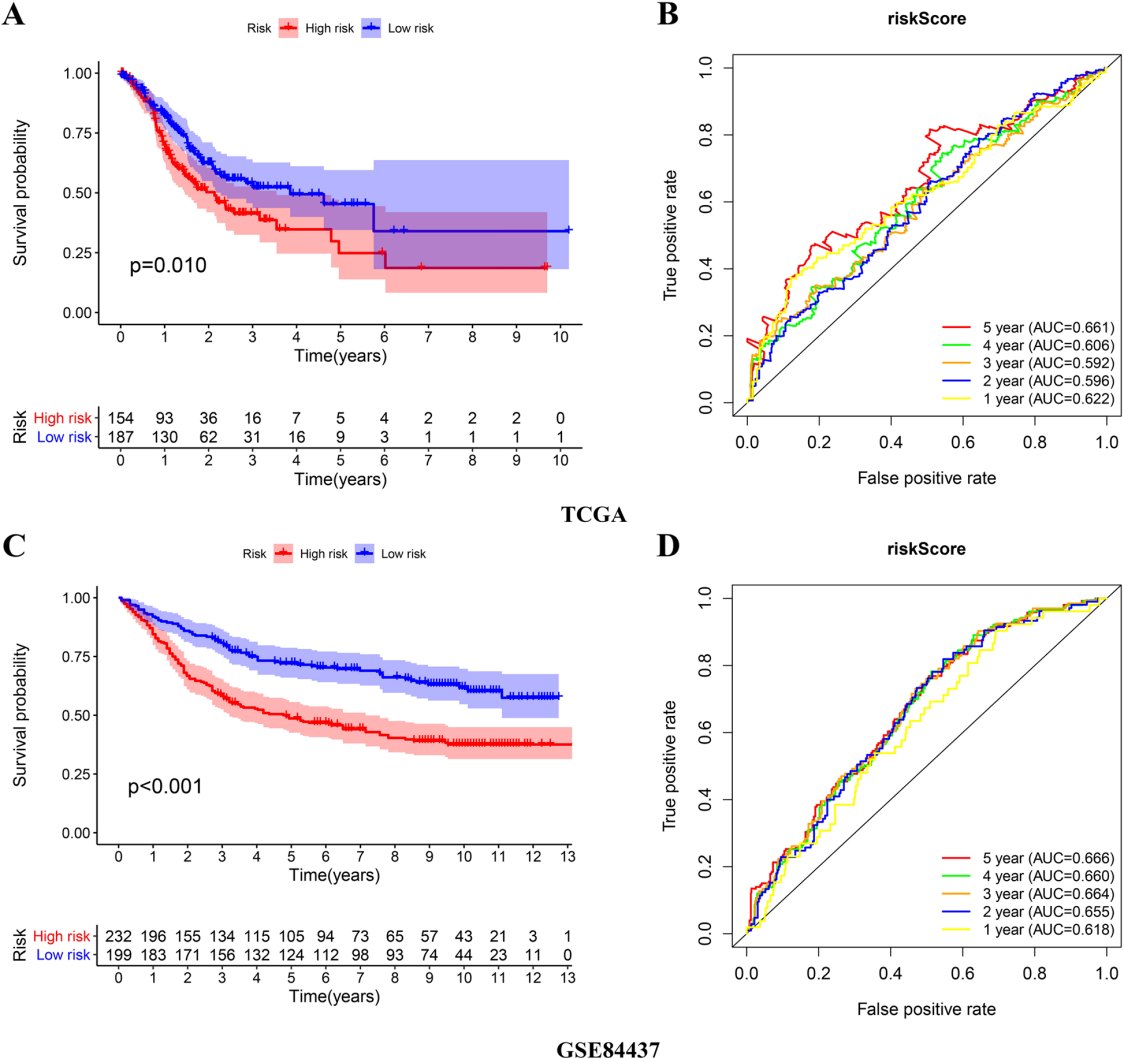
Internal validation of the risk score in the TCGA and GSE84437 cohort. **A**, **B** The Kaplan–Meier survival analysis and the time-dependent ROC analysis of the signature for predicting the OS of patients in the TCGA cohort. **C**, **D** The Kaplan–Meier survival analysis and the time-dependent ROC analysis of the signature for predicting the OS of patients in the GSE84437 cohort

The patients in the high risk group exhibited significantly lower OS than the low risk patients in both TCGA and GSE84437 cohort (Fig. 6A, C). The AUC values of the risk score predicting 5-year survival rate of GC patients in TCGA and GSE84437 cohort were 0.661 and 0.666, respectively (Fig. 6B, D).

### Exploration the relationship between the prognostic signature and the immune cell infiltration characterization

**Fig. 7:**
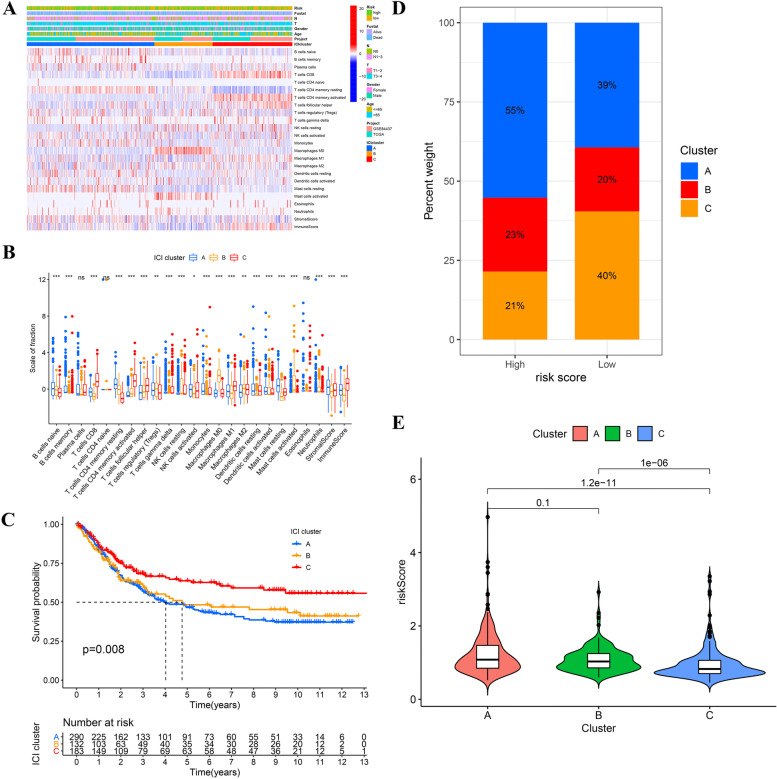
The relationship between the risk score and the immune cell infiltration characterization. **A**, **B** The heatmap and boxplot of immune cell infiltration for the three subtypes. **C** The Kaplan–Meier survival analysis the three subtypes. **D** The barplot of proportions of the three subtypes in high- and low-risk groups. **E** The vioplot of the risk score difference among the three subtypes

According to the infiltration of 22 kinds of immune cells, the patients were clustered into three subtypes (Fig. 7A, B). There were significant differences in the prognosis of different subtypes; cluster A had the worst prognosis, while cluster C had the best prognosis (Fig. 7C). The proportion of cluster A in high-risk group was significantly higher than that in low-risk group (Fig. 7D). The risk score of cluster A and cluster B was significantly higher than that of cluster C (Fig. 7E).

### External validation of the prognostic signature in four independent cohorts

**Fig. 8:**
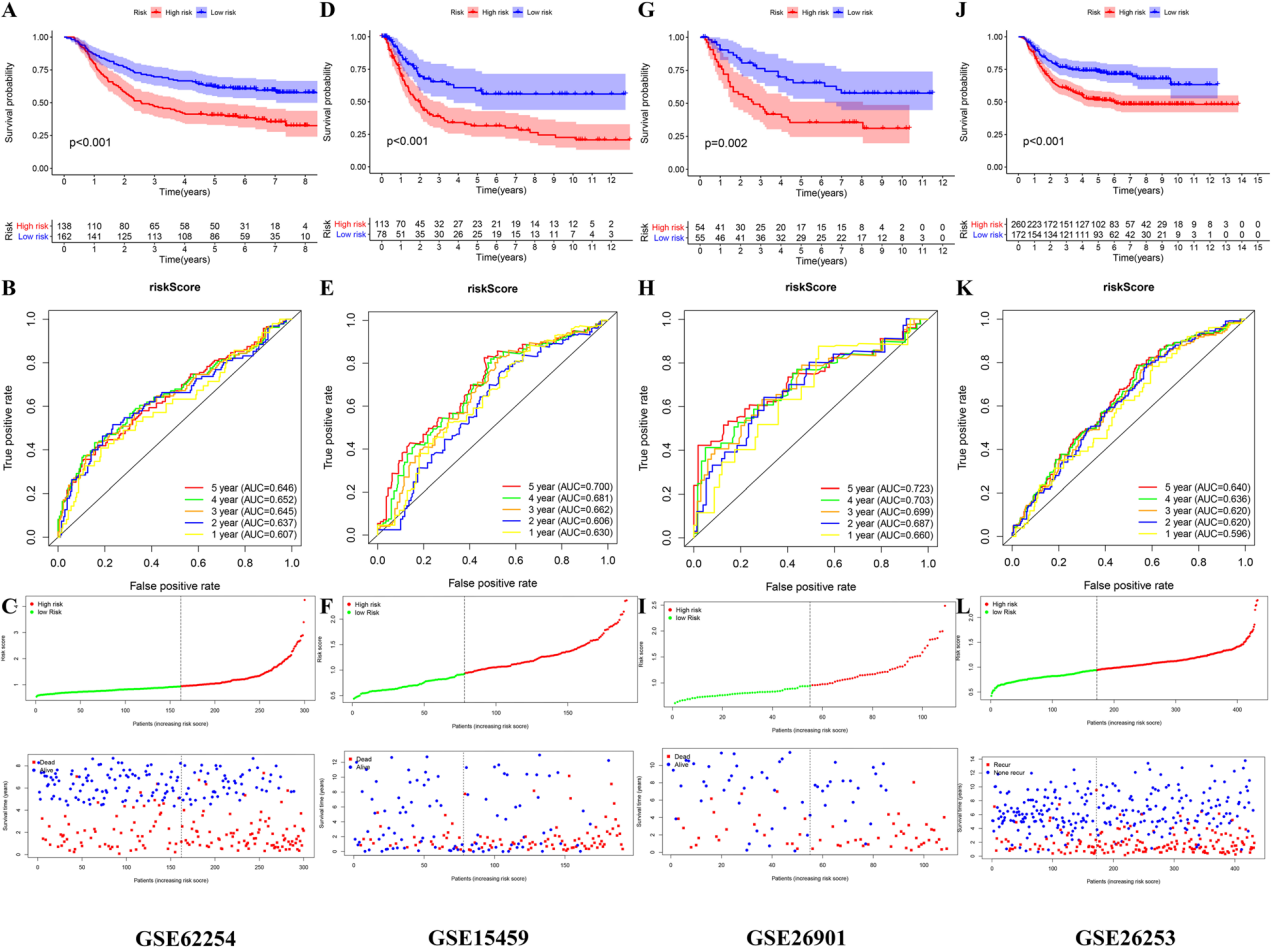
External validation of the prognostic model. **A**, **B** The Kaplan–Meier survival analysis and the time-dependent ROC analysis of the signature for predicting the OS of patients in the GSE62254 cohort. **C** The distribution of risk score, and the survival status of patients in in the GSE84437 cohort. **D**, **E** The Kaplan–Meier survival analysis and the time-dependent ROC analysis of the signature for predicting the OS of patients in the GSE15459 cohort. **F** The distribution of risk score, and the survival status of patients in in the GSE15459 cohort. **G**, **H** The Kaplan–Meier survival analysis and the time-dependent ROC analysis of the signature for predicting the OS of patients in the GSE26901 cohort. **I** The distribution of risk score, and the survival status of patients in in the GSE26901 cohort. **J**, **K** The Kaplan–Meier survival analysis and the time-dependent ROC analysis of the signature for predicting the RFS of patients in the GSE26253 cohort. **L** The distribution of risk score, and the recurrent status of patients in in the GSE26253 cohort

The high risk group had significantly lower overall survival (OS) and recurrence-free survival (RFS) in the four independent cohorts (Fig. 8A, D, G, J). The AUC values of the risk score predicting 5-year survival rate of GC patients in the GSE62254, GSE15459, and GSE26901 cohorts were 0.646, 0.700, and 0.723, respectively (Fig. 8B, E, H). The AUC values of the risk score predicting 5-year RFS of GC patients in the GSE26253 cohort was 0.640 (Fig. 7K). The high-risk group had a higher risk of death and recurrence (Fig. 8C, F, I, L).

## Discussion

Tumor heterogeneity is one of the characteristics of malignant, and it is also a major challenge for human beings to overcome tumors [24]. Clinically, some blood indexes, such as lymphocyte/monocyte ratio, neutrophil/lymphocyte ratio, and platelet/lymphocyte ratio, are of great significance for monitoring the progress and prognosis of many diseases, including virus infection, autoimmune diseases, and so on, because of their advantages of dynamic and real-time detection [25]. Although simple blood indicators can provide reference for monitoring cancer treatment and guiding prognosis to a certain extent, they play a very limited role in evaluating tumor heterogeneity. The proposal of tumor microenvironment (TME) provides new insights for human beings to better understand the heterogeneity of tumors [26]. Stromal cells are one of the most abundant and critical components of TME, which could affect many aspects of tumor progression by remodeling extracellular matrix, such as proliferation, invasion, angiogenesis, and metastasis [27]. According to the latest viewpoint, stromal cells can also secrete a variety of cytokines and chemokines or indirectly interact with immune cells in tumor microenvironment to regulate the transformation, infiltration, and function of immune cells, making TME in an immunosuppressive state [28]. To further clarify the mechanism of stromal cells in tumor immune regulation is expected to find a new strategy for the treatment of cancer.

Gastric cancer (GC), as a common digestive system tumor, is the fifth largest malignant tumor in the world and the third leading cause of cancer death [29]. Surgical resection, radiotherapy, and chemotherapy are still the main treatment methods, but the effect is not ideal [12]. In recent years, experts in the field of GC research have pointed out that parameters reflecting individual immune status, such as preoperative systemic immune-inflammation index (SII) [30], Follistatin-like 1 (FSTL1) [31], immune T cell subsets (including CD3+, CD4+, CD8+, CD4+/CD8+ ratio, NK Cells) [32], the preoperative and the postoperative neutrophil-to-lymphocyte ratios (NLR) [33–36], and platelet-lymphocyte ratio (PLR) [37], may be useful prognostic indicators of GC. Previously studies have shown that immune-related genes can effectively predict the prognosis of patients with colonic adenocarcinoma [38], but the relationship between immune-related genes and the prognosis of GC has not been fully clarified. Considering the occult pathogenesis of GC, its recurrence and metastasis are closely related to tumor microenvironment; further understanding of the role of stromal cells in the immune regulation of GC is expected to improve the current treatment strategy for GC.

In two independent cohorts with a sample size of more than 300 (TCGA, GSE84437), we observed poor prognosis in patients with high stromal cell abundance, which further confirmed the negative effect of stromal cells on the prognosis of GC. By comparing the expression of immune genome in patients with high stromal score and low stromal score, we obtained a total of 167 common differential genes in two independent cohorts. TCGA and GSE84437 were intergrated into a large sample cohort (*n*=772) to analyze the prognostic value of the intersection genes. After univariate cox regression analysis, LASSO regression analysis, and multivariate cox regression analysis, a six-gene risk score was established.

Through internal validation (TCGA, *n*=341; GSE84437, *n*=431) and external validation (GSE26253, *n*=432; GSE62254, *n*=300; GSE15459, *n*=191; GSE26901, *n*=109), we found that risk score had good predictive ability for predicting 5-year overall survival (OS) rate and 5-year recurrence-free survival (RFS) rate of GC patients. The risk of death and recurrence increased with the increase of risk score, indicating that risk score played an important role in the progression of GC. Given the heterogeneity of GC, the robustness of risk score may be an effective auxiliary tool to evaluate the prognosis of GC.

In recent years, judging the prognosis of tumors by immunophenotype has become a research hotspot [39–41], but similar studies in GC are still very rare. We attempted to reveal the underlying mechanism of the risk score by performed the cluster analysis of immune cell infiltration (ICI). The patients were divided into three subtypes according to the difference of ICI. There were significant differences in the prognosis of different subtypes. Cluster A, the worst prognosis group, accounted for more than half of the high-risk group; the risk score of cluster C subtype with the best prognosis was significantly lower than that of the other two subtypes. Therefore, the difference of immune infiltration may be an important reason leading the difference of prognosis between different risk groups.

Stromal cells in tumor microenvironment have a variety of tumor-promoting functions and promote immune escape through a variety of mechanisms, but there are no clinical inhibitors for tumor stromal cells. In intrahepatic cholangiocarcinoma, m2 polarized tumor-associated macrophages promote epithelial-mesenchymal transformation by activating AKT3/PRAS40 signaling pathway [42]. In colorectal cancer, propofol inhibits cell proliferation and metastasis by regulating miR-124-3p.1/AKT3 [43]. In ovarian cancer, the overexpression of AKT3 could promote the proliferation and migration of cancer cells [44]. However, the specific role of AKT3 in the progression of gastric cancer is unknown. Apolipoprotein D (APOD) is a protein regulated by androgen and estrogen. The increased expression of APOD could predict poor prognosis in patients with breast cancer independent of the expression of ER α and AR [45]. In patients with mantle cell lymphoma, patients with high expression of FAM19A5 are more likely to relapse or die [46]. LTBP3 is a new cancer target protein, which has a unique function in the regulation of vascularization of tumor cells dependent on angiogenesis [47]. Nephroblastoma overexpressed protein (NOV), an early member of the CCN family, has recently been thought to be involved in a series of inflammatory processes such as wound healing, alveolar epithelial cell inflammation, cancer metastasis, and macrophage foam cell formation [48]. NOX4 played an important role in maintaining the phenotype of immunosuppressive cancer-associated fibroblasts (CAF) in tumors; immunotherapy could be enhanced by suppressing NOX4 to overcome CD8T cell rejection mediated by CAFs [49]. The above six molecules may be expected to become potential targets for targeted therapy of stromal cells.

This study provided new insights into the prognosis assessment of patients with gastric cancer by analyzing the gene expression profiles and prognostic data of 1804 patients from six centers. This is the largest scale retrospective study we have ever known about the development of a prognostic model for gastric cancer, but it does not mean that multicenter prospective studies are no longer necessary. The effect of stromal cells on tumor is not only limited to immune regulation, but also includes metabolic reprogramming and many other aspects, which need to be further explored. In addition, there are few reports on the specific role of the above six genes in the development of gastric cancer, which need to be further clarified.

## Conclusion

This study established and validated a robust prognostic model for gastric cancer by intergrated analysis of 1804 samples of 6 centers, and its mechanism was explored in combination with immune cell infiltration characterization.

## Data Availability

The datasets analyzed for this study were obtained from The Cancer Genome Atlas (TCGA, https://portal.gdc.cancer.gov/) and Gene Expression Omnibus (GEO, https://www.ncbi.nlm.nih.gov/geo/).

## Abbreviations

GC: Gastric cancer
TCGA: The Cancer Genome Atlas
GEO: Gene Expression Ominibus
TME: Tumor microenvironment
ESTIMATE: Estimation of STromal and Immune cells in MAlignant Tumor tissues using Expression data
DEIRGs: Differential expressed immune-related genes
SAIRGs: StromalScore Associated Immune-Related Genes
LASSO: Least absolute shrinkage and selection operator
ROC: Receiver operating characteristic
AUC: Area under curve
OS: Overall survival
RFS: Recurrence-free survival
ICI: Immune cell infiltration
